# Minimizing Atrioesophageal Fistula Risk After AF Ablation: Risk Factors, Prevention, and Emerging Mini‐Thermal Technology

**DOI:** 10.1111/pace.70037

**Published:** 2025-09-02

**Authors:** Muhammed Ibrahim Erbay, Esedullah Yağlı, Tasha Phillips‐Wilson, Arda Çeviker, Henry D. Huang, Joseph E. Marine, Kıvanç Yalın

**Affiliations:** ^1^ Cerrahpasa Faculty of Medicine Istanbul Cerrahpasa University Istanbul Turkiye; ^2^ University of Michigan School of Public Health Ann Arbor Michigan USA; ^3^ Cerrahpasa Faculty of Medicine Department of Cardiology Istanbul Cerrahpasa University Istanbul Turkiye; ^4^ Division of Cardiology Department of Medicine Rush University Medical Center Chicago Illinois USA; ^5^ Division of Cardiology Department of Medicine Johns Hopkins University School of Medicine Baltimore Maryland USA

**Keywords:** atrial esophageal fistula, atrial fibrillation ablation, catheter ablation complications, cryo‐balloon ablation, esophageal thermal injury, pulsed‐field ablation, radiofrequency ablation

## Abstract

Atrial esophageal fistula (AEF) is a rare but life‐threatening complication of atrial fibrillation (AF) ablation, linked to thermal injuries by conventional radiofrequency (RF) and cryo‐balloon (CB) ablation techniques. AEF risk can be mitigated by considering several measures such as tailored power settings of ablation technique, mechanical displacement of esophagus, esophageal cooling, and alternative ablative techniques and energy sources. We review the current knowledge regarding AEF and esophageal thermal injuries as well as discussing the current research regarding a novel none‐to‐minimally thermal, myocardial tissue‐selective modality known as pulsed‐field ablation (PFA) which may mitigate such risks. By inducing irreversible electroporation, PFA reduces thermal injury and demonstrates improved safety profiles, as evidenced by recent meta‐analyses reporting zero esophageal injury and AEF cases. Additionally, the integration of 3D mapping systems with PFA has enhanced its procedural precision and accuracy while lowering the radiation exposure. Despite these advances, challenges such as standardizing anesthesia protocols and tailoring energy settings remain. Our review suggests that PFA may reduce the risk of AEF from catheter ablation of AF. While early outcomes of PFA are encouraging, it is important to recognize that preliminary data may not always be predictive of long‐term AEF formation risk, as demonstrated by earlier experiences with CB ablation. Although PFA may reduce the risk of any aberrant thermal injuries, recent studies report significant increase in collateral damage including hemolysis, exaggerated troponin leak and coronary vasospasms. PFA should be used with caution in patients with hemolytic anemia or renal dysfunction, as they may experience more pronounced effects. Future long‐term outcome studies should provide more information on possible adverse outcomes with PFA as well as tailoring the power settings of PFA.

**Abbreviations**
AEF: atrial esophageal fistula
AF: atrial fibrillation
CB: cryo‐balloon ablation
CT: computed tomography
ENPT: endoscopic negative pressure therapy
EP: electrophysiology
Fcal: fecal calprotectin
HPSD: high power shorter duration
IRE: irreversible electroporation
LA: left atrium
LET: luminal esophageal temperature
LPLD: low power longer duration
MDTs: multidisciplinary care teams
PFA: pulsed field ablation
PVI: pulmonary vein isolations
RF: radiofrequency
TEE: transesophageal echocardiogram

## 1 Introduction

Atrial fibrillation (AF) ablation procedures typically involve electrical isolation of the pulmonary veins within the left atrial chamber of the heart which lies adjacent to the esophagus. With this anatomical proximity, atrioesophageal fistula (AEF) is a rare complication with high mortality and morbidity associated with ablative procedures intended to mitigate the arrhythmia. The condition can be suspected from a wide range of symptoms, including dysphagia, fever, constitutional, or neurological symptoms due to life‐threatening air embolism, making a timely diagnosis and intervention crucial [1]. Despite its severity, AEF remains underrecognized and understudied in clinical practice and research due to its rarity.

Conventional AF ablation modalities, including radiofrequency (RF) and cryo‐balloon (CB) ablation, create injury to myocardial tissue via inflammation that is caused by small burns or freezes and the subsequent healing process. This antiarrhythmic mechanism carries the risk of esophageal thermal injury, which can cause a subsequent fistula between the left atrium (LA) and proximal esophageal tissue via necrosis, leading to the formation of AEF [2]. The spectrum of esophageal injury after ablation for atrial fibrillation ranges from mild mucosal injury to esophageal perforation and fistula formation [3].

Considering these risks, alternative methods and promising techniques have been proposed, including pulsed‐field ablation (PFA). PFA as an ablation technique relies on the delivery of short‐duration, high‐amplitude electrical fields that create irreversible pores within selective cardiomyocytes, leading to cell apoptosis. Theoretically, PFA is a safer technique than conventional ablation techniques due to being a “minimally‐thermal” choice and its myocardial‐tissue selectivity. The electrical field amplitude level for driving myocardial cell apoptosis is believed to be different from esophageal tissue, nervous system, and pulmonary veins, which were previously at risk of thermal injury.

## 2 Incidence and Risk Factors of AEF

Incidence of AEF is difficult to assess due to its rarity, although there is a reported incidence rate of 0.02%–0.25% after a variety of associated ablation modalities. It should also be noted that many incidences remain undiagnosed, obscuring the actual incidence levels. In addition to its rarity, AEF is also well‐known to be a potentially fatal entity, with a mortality rate of 80%, particularly when recognition is delayed. In contrast, the survival rate is 64.7% after surgical interventions and only 2.8% with medical treatment alone [4].

New technologies for AF catheter ablation are continuously being developed, and thus, the risk of AEF formation may differ according to the technique used during ablation procedures. A multi‐center retrospective study with a total of 14,224 patients showed that there were no incidences of AEF for the equivalent period in cohorts for which active esophageal cooling has been used [5, 6].

AEF is more prevalent in men compared to women, which is attributed in part to men requiring more RF ablations and additional ablation lesions compared to women [7, 8]. Although the exact mechanism of AEF is not completely understood, it is believed that AEFs typically start from esophageal ulcerations, which are the result of esophageal mucosal injury during the ablation of the LA [9, 10]. The anatomical proximity of the esophagus to ablation sites causes the luminal temperature to rise, and the thermal energy damages the vascular structure, branches of the vagus, and lymph nodes in the area, which may be risk factors of AEF formation [11]. In a study by Halm et al., higher luminal esophageal temperature (LET) was associated with esophageal injury, but the authors did not find any esophageal lesions with peak temperature below 41°C, so it could be inferred that LET >41°C contributes as an additional risk factor [12]. Risk between individuals may be heterogeneous based on anatomical factors that can vary with every person, such as LA‐esophagus distance, width of area between esophagus and LA, thickness of adipose tissue layer between the LA and esophagus, and left atrial size [13, 14].

**FIGURE 1:**
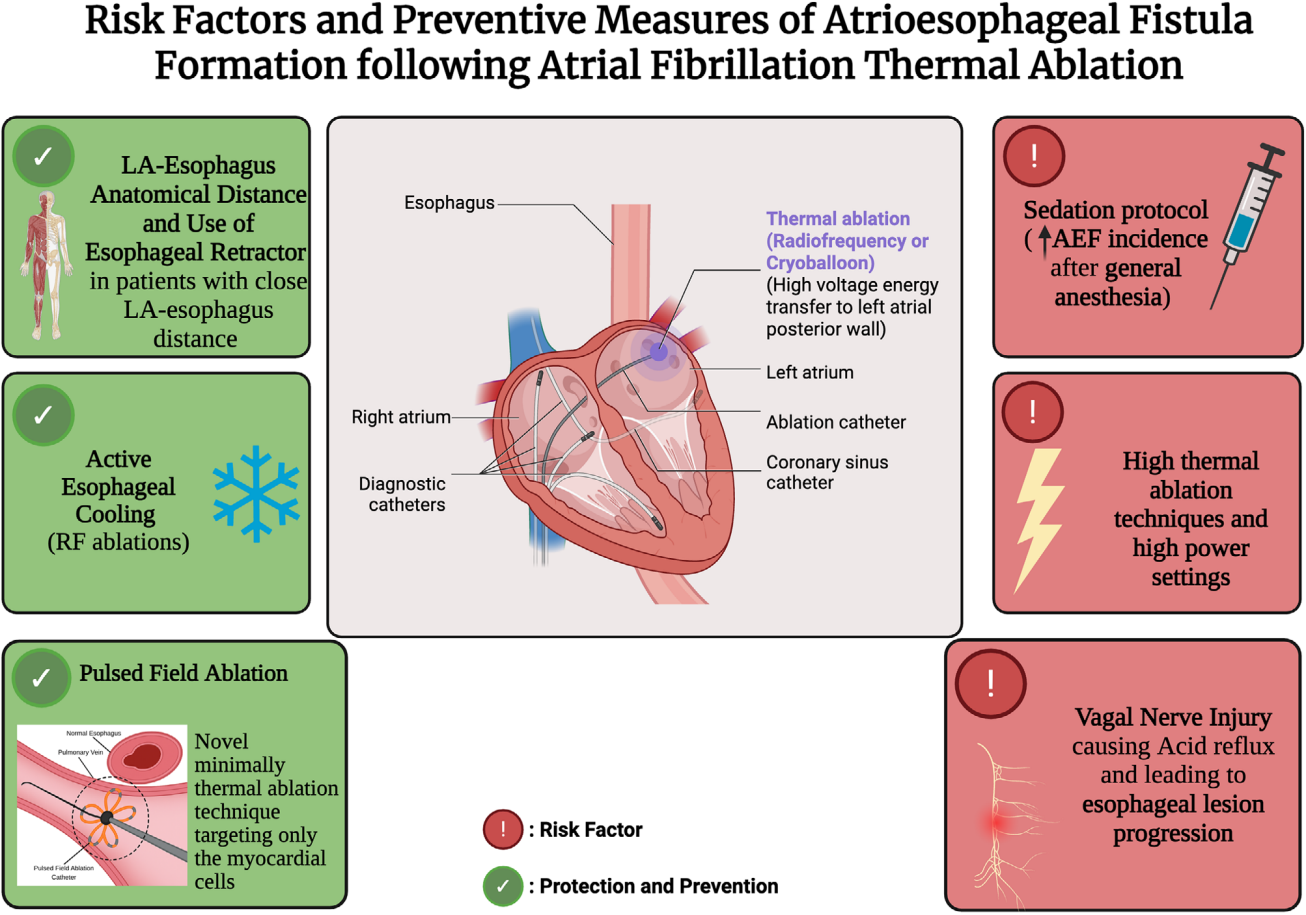
Risk factors and preventive measures of atrioesophageal fistula formation following atrial fibrillation ablation. Created with BioRender. [Colour figure can be viewed at wileyonlinelibrary.com]

Sedation type might also affect the incidence of AEF. In one study, the incidence of esophageal injury was found to be 48% in the general anesthesia group and only 4% in the local anesthesia group. This difference might be due to less esophageal motility, absence of swallowing, and esophageal fixation of the nasogastric tube in the general anesthesia group [15]. Active esophageal cooling devices that circulate cold water in a closed circuit are more effective than LET monitoring in maintaining stable periesophageal temperatures and reducing the incidence of premature ablation cessation (Figure 1). LET monitoring has several drawbacks: (1) it does not measure periesophageal or esophageal wall temperatures directly, and (2) its sensitivity to temperature changes is highly dependent on the proximity of the sensor to the ablation source. Therefore, the reliability of LET as a protective measure during ablation procedures remains questionable and should be clearly acknowledged [16, 17]. It is also important to note that active esophageal cooling is an effective tool in reducing esophageal lesions after RF ablation, decreasing fluoroscopy levels, and arrhythmia recurrence. In contrast, esophageal warming in CB ablation does not provide the same value and is not effective in decreasing thermal lesions [18].

## 3 Pathophysiology

The anatomical relationship of the esophagus to the LA is key to understanding the pathophysiology of AEF. The esophagus is posteriorly located in relation to the LA heading down to the stomach in a bow‐like trace. AF catheter ablation most commonly involves circumferential ablations of the four pulmonary vein antral area and sometimes additional lesions on the posterior wall inside the box lesion set [19, 20]. During AF catheter ablation, different methods are used for the ablation of arrhythmia triggers that interrupt normal electrical pathways. Even though AEF formation is more common following RF, CB ablations also cause esophageal thermal lesions and AEF [21].

RF ablation destroys cardiac tissue in 2 phases: (1) resistive heating, which transfers a high amount of heat energy in ultra‐rapid duration, and (2) conductive heating, which leads to much of the lesion size through an increase in surrounding tissue temperature. Further, RF energy is associated with thermal latency, which results in a continued increase or delayed decrease in tissue temperature even after the operator has stopped RF application. The rise of temperature within the esophagus occludes end‐arterioles, and thermal injury leads to esophageal mucosal ischemia [22]. Every 1°C increase in endoluminal temperature increases the odds of an esophageal lesion by 1.4 [23]. As mentioned above, a rise in LET depends on anatomical factors such as LA‐esophagus distance, thickness of fat layer between esophagus and atrium, width of interaction surface, and esophageal motility. Even though CB ablation was initially expected to prevent overheating due to its nature, later studies demonstrated that CB ablations are also associated with AEF formation, esophageal thermal injuries, and phrenic nerve injuries [21]. The left inferior pulmonary vein is reported to be the most common culprit in AEF formation after CB ablations [24, 25].

Heat‐induced esophageal damage causes ulceration, and esophageal ulceration is considered a precursor to the AEF. The damage caused by esophageal injury can be exacerbated by pre‐existing gastroesophageal reflux or damage to periesophageal nerves during ablation. Ablation therapy may also cause gastroesophageal reflux due to vagus nerve injury (VNI), since the cranial nerve controls peristalsis of the esophagus, tonus of sphincters, and antral motility [23, 26]. Therefore, periesophageal vagal plexus injury may cause gastric hypomotility, pyloric spasm, and lower esophageal sphincter dysfunction, thereby exacerbating the esophageal injury [27].

SEPIA study showed us that VNI after pulmonary vein isolations (PVI) occurs in almost one in third of patients (27/85) and this is observed more frequently in patients with pre‐existing esophagitis [25]. Although this is not fully overlapped with endoscopic findings within 2 days post‐PVI, the study is important to understand the potential impact of VNI‐associated acidic reflux, adding to the esophageal lesion progression to fistula. Furthermore, the findings correlate with the 2015 Atrial Fibrillation Gut study, which showed that AF ablation may cause functional upper gastrointestinal impairment [28]. Esophageal injury following radiofrequency (RF) ablation presents a spectrum of complications, ranging from esophageal ulcerations and perforations to esophago‐pericardial fistula and AEF. This progression appears to follow a gradual pathogenesis, beginning with ulcerations and potentially culminating in AEF formation [29]. Unlike esophageal perforation, atrial perforation alone is rare; the fistula typically allows leakage from the esophagus into the left atrium rather than the reverse. This pathological connection between the esophagus and heart can lead to severe neurological consequences, including air and food emboli, stroke, and meningitis, which significantly increase morbidity and mortality [30, 31].

### 3.1 Understanding Mathematical Equations Behind RF Catheter Ablation

RF ablation is a widely deployed ablation technique that, in simple terms, transfers heat energy to ablation sites. To mitigate esophageal thermal injury leading to AEF formation while achieving successful ablation endpoints, it is crucial to adjust the transferred energy and its ablation duration. Several mathematical models are used to understand the heat transfer within blood flow and to determine sufficient tissue damage over time. Continuous blood flow carries the heat away from the ablation zone. Pennes’ equation calculates how heat moves through circulation, considering the blood flow, heat applied by the ablation device, and the heat lost to surrounding tissue. Wave equations describe how electromagnetic waves are delivered within the ablation zone. Arrhenius equation helps with the accurate measurement of the temperature dependence of tissues to prevent any additional thermal injuries beyond ablation zones. In other words, the Arrhenius equation tells us how quickly a cell dies with a certain level of temperature and the required time for a successful ablation [32]. The equation is:

$$k=Ae^{\frac{-Ea}{RT}}$$

*k* = rate constant, *A* = pre‐exponential factor, *E_a_* = activation energy (in the same units as R*T), *R* = universal gas constant, *T* = absolute temperature (in Kelvin).

## 4 Diagnosis and Treatment

### 4.1 Suspicion of AEF Injury

Most AEF cases tend to present asymptomatically until the formation of a blown‐out fistula. This can lead to late diagnosis and worse prognosis, making the suspicion of AEF a valuable step in the diagnosis of AEF. A raised index of suspicion for AEF is warranted in patients who exhibit acute symptoms of fever, chest pain, odynophagia, neurological signs, dyspnea, constitutional symptoms, and hematemesis within days or weeks of an atrial fibrillation ablation [2, 33] (Table 1). The POTTER‐AF study showed that time‐to‐first symptoms occurred with a median time of 18 (7.75–25) days, while the diagnosis was made after a median 21 days (15–29.5) of the procedure (Table 2). Most common complications include severe sepsis/septic shock (57.9%), coma (46.7%), stroke/cerebral hemorrhage (23.4%), cardiac arrest (18.7%), gastrointestinal bleeding (16.8%), and cardiac tamponade (11.2%) (Table 3). Suspicion is also high in patients who develop post‐ablation esophageal ulcers, which precede the development of an AEF [23]. In these instances, a prompt diagnosis and subsequent intervention are warranted to prevent iatrogenic‐related mortality, which is highly likely without emergent treatment [34].

**TABLE 1:** Clinical presentation of patients with AEF after Afib ablation by percentage [2].

Clinical presentation	Reported symptoms (% of patients)
Fever	59.30%
Pain/Odynophagia	54.20%
Neurological signs	44.10%
Others	62.30%
Dyspnea	5.10%
Nausea	4.20%
Syncope	3.40%
Cough	2.50%
Atrial Fibrillation	2.50%
Hematemesis/Confusion/Head & Neck Pain/Vomiting/STEMI	1.7% (each complication)
Aphasia	0.80%

**TABLE 2:** Postprocedural time to first symptoms and postprocedural median diagnosis time [2].

Esophageal fistula formation	Median time
Time to first symptoms	18 days (7.8–25 days)
Diagnosis post‐procedural	21 days (15.0–29.5 days)

**TABLE 3:** Most common AEF complications and their percentages [2].

Complications	Percentage (%)
Severe sepsis/septic shock	57.90%
Coma	46.70%
Stroke/cerebral hemorrhage	23.40%
Cardiac arrest	18.70%
Gastrointestinal bleeding	16.80%
Cardiac tamponade	11.20%
Others	29.00%

Each atrial fibrillation ablation technique reports associated complications and carry a potential of AEF development [23]. Therefore, diagnosis of an AEF can be made by assessing a patient's recent clinical history along with their presenting cardiac, gastrointestinal, and neurologic symptoms, supported with chest imaging [35].

Diagnostic and confirmatory findings are obtained with computed tomography (CT) of the chest, including oral and intravenous contrast to improve visualization of the heart and esophagus [23]. While performing CT is an important step in the diagnosis of AEF, soluble contrast agents used during the procedure must be water‐soluble [36]. Magnetic resonance imaging (MRI) of the esophagus, particularly late gadolinium enhancement (LGE), is also effective for assessing esophageal thermal injury [37]. Helpful in ruling out AEF in patients with allergies to contrast is dynamic MRI hydrography [38], a novel and alternative imaging technique [26, 36]. However, useful in esophageal thermal injury identification, imaging modalities that should generally be avoided when AEF is suspected include transesophageal echocardiogram (TEE) or esophageal endoscopy, as this procedure notably carries a high risk of complications, including fatalities secondary to acute stroke and air embolism with insufflation [23, 39, 40].

In the evaluation of suspected AEF post‐AF ablation, fecal calprotectin (Fcal), a calcium and zinc‐bound protein found in neutrophils, detects gastrointestinal inflammation. This acute phase protein and biomarker play a predictive role in AEF development as a cut‐off value of 125 µg/g is indicative of esophageal injury, thus indirectly increasing the risk of an AEF complication in patients [41, 42]. However, Fcal is not specific to esophageal thermal injury and can be elevated in numerous GI tract inflammation and pathologies such as inflammatory bowel disease, infections, and malignancies. An FDA‐approved proactive esophageal cooling method may be performed to prevent any possible RF ablation‐related esophageal injury [43, 44].

Multidisciplinary care teams (MDTs) bring clarity to the collaborative tasks of inpatient physicians, nurses, pharmacists, social workers, and rehabilitation teams dedicated to their expertise and specific duty to the patient [45]. These teams take into account not only life‐sustaining measures but also the consideration of healthcare ethics, including advanced care directives as necessary, given the increased risk of mortality and long‐term morbidity in AEF [46]. Additionally, MDTs with good communication and team cohesiveness improve overall patient safety, satisfaction, and adherence to medical plans [47]. When well structured, these MDTs improve resource optimization by employing quality improvement initiatives and a reduction in healthcare costs by way of decreased medical errors and shorter hospital stays [44, 48]. Collectively, when multidisciplinary healthcare teams function as a unit, patients receive more comprehensive assessments and plans which significantly improve the health outcomes and survival rates of AEF patients [49]. The approach and impact multidisciplinary teams have on care quality reflect the institution or clinical program they represent and the team leaders.

### 4.2 Endoscopic Negative Pressure Therapy (ENPT) Can Be Used to Heal Thermal Lesions After AF Ablation Procedures

Deployment of ENPT is a novel technique that can be used for deeper AF ablation lesions, resulting in a significant decrease in the diameter and depth of the lesions [50]. ENPT has been previously used for expediting wound healing for the treatment of transmural injuries. Its mechanisms of effectiveness include macro and micro deformation, angiogenesis, exudate control, and bacterial clearance. This experimental technique can help reverse the thermal lesions and prevent their progression.

## 5 Prognosis and Outcomes

Post‐ablation AEF is a life‐threatening complication with remarkably high mortality rates, with and without intervention [26, 51]. Factors that influence the prognosis of a post‐AEF diagnosis include specific patient demographics (age, gender), clinical features (primary vs. secondary AEF), and diagnostic timing [23, 35, 52]. If diagnosis and treatment are delayed, the risk of near‐term mortality increases substantially. Additionally, patients with comorbidities, including hypertension, COPD, diabetes, and compromised renal function, have a worse prognosis than other patients. However, patients with severe acute presentations with hemorrhagic shock, chest pain, dysphagia, and abdominal pain have an even worse prognosis as these baseline deficits exacerbate the injury and increase the risk for post‐treatment complications [52].

Mortality rates decrease with treatment expediency; therefore, urgent AEF interventions typically receive a better prognosis and experience more favorable results after surgical repair [53]. Other predictors of mortality include increased patient age, existing comorbidities, and gastrointestinal bleeding [35].

The complications and mortality rates of AEF interventions are high, but they vary based on the treatment method [54]. Patients treated for AEF with surgical repair have mortality rates that range from 30% to 35%, as opposed to greater than 70% with medical management alone or esophageal stenting [23, 35, 55]. In contrast, patients who receive conservative to no therapeutic intervention have the worst prognosis—a mortality rate approaching 100% because AEF is usually fatal when left untreated, and patients typically succumb to hemorrhagic shock, sepsis, or multiorgan failure [52].

The long‐term status of patients with successful AEF interventions is not yet known due to the elevated risk of mortality [26]. However, patients with long‐term complications of post‐intervention‐AEF include sepsis, multiorgan failure and hemorrhagic shock, cardiac arrest, and neurologic injury [26].

Follow‐up considerations post AEF should include a noninvasive CT scan‐monitoring affected cardiac and esophageal tissue for appropriate tissue healing and rehabilitation [56].

## 6 Preventive Strategies

Although AEF formation occurs very rarely after AF catheter ablation cases, it can have delayed mortal consequences with a poor prognosis [57, 58]. Therefore, it is crucial to identify and avoid the factors that can lead to AEF formation. Detailed risk assessment of patients and application of appropriate procedural precautions leading to successful PVI in higher‐risk procedures can optimize AEF risk management [59].

### 6.1 Procedural Precautions

Bodziock et al. have demonstrated well‐structured periprocedural AEF preventive measures taking into consideration low risk, cost‐effectiveness, and improved outcomes. These key factors that affect AEF risk during AF catheter ablation include sedation type, ablation modality and power settings, esophageal temperature monitoring, mechanical esophageal deviation, and pharmacologic prophylaxis [60].

#### 6.1.1 Sedation

Conscious sedation (using fentanyl or midazolam) may carry a lower risk of AEF than general anesthesia, reducing esophageal contact and tissue damage. Additionally, general anesthesia lowers esophageal motility, which causes patients to be incapable of alerting the operator to any discomfort [60]. Pang et al. (2022), however, have found that GA/deep sedation decreases the recurrence of persistent and paradoxical AF without altering the incidence rate compared to conscious sedation [61]. Sedation type should be chosen based on the patient's characteristics and preferences, and availability of an anesthetist [34, 62].

#### 6.1.2 Power Settings

Chieng et al. (2023) in the Hi‐Lo HEAT Trial showed that choosing high power (40 W vs. 25 W), shorter duration (7–11 s) (HPSD) ablation decreased esophageal thermal injury when compared to low power, longer duration (LPLD) RF ablation [63]. AF recurrence was also lower in the HPSD group (*p* = 0.04), which can be attributed to wider, shallower, and overlapping lesions that support contiguity of the ablation lines without allowing late‐gap formation. Notably, with a *p* value of 0.43, there were no significant differences in maximal esophageal temperature (HPSD 38.6°C vs. LPLD 38.7°C), or the amount of lesions (HPSD 1.5 vs. LPLD 2, *p* = 0.93) [63, 64]. However, HPSD creates shallower lesions which poses a smaller risk of thermal injury to the surrounding tissue including the esophagus [63, 65]. Pranata et al. (2023) showed that using very HPSD (VHPSD—(70–90 W/4–7 s)) resulted in a lower AF recurrence, but similar low complication rates compared to conventional ablation (30–40 W/>20 s, 50 W/7–11 s). In brief, HPSD (∼50 W), but not VHPSD (∼70–80 W), has lowered complication rates including esophageal thermal injuries and both VHPSD and HPSD shortened the overall procedural and RF ablation times [63, 66].

#### 6.1.3 Contact Force‐Sensing Catheters

The ability of a catheter to measure the pressure applied to the endocardium, otherwise known as contact force (CF)‐sensing catheters, may also reduce the risk of AEF formation. Zhang et al. (2019) in the RESCUE‐AF Trial have shown that CF‐sensing catheters restricted to <20 g minimized the risk of esophageal injury as compared to non‐sensing catheters [67]. Measuring the applied force on the posterior left atrial wall allows operators to have more control over mitigating the risk of esophageal pressure injury. CF‐sensing catheters also optimize the formation of sufficient lesions, increasing procedural success by decreasing recurrence and complications [68]. Thus, CF‐sensing catheters are widely accepted as a safer and an effective tool by offering improved lesion formation and minimizing the risk of esophageal injury.

#### 6.1.4 Esophageal Temperature Monitoring, Active Esophageal Cooling, and Temperature Control Devices Help Warn Operators

Esophageal temperature monitoring may provide insight into the risk of thermal injury, allowing titration of energy based on the patient [69]. However, use of LET‐only may result in premature cessation of RF energy deposition, leading to a higher incidence of re‐do procedures and freedom from arrhythmia, and also increase fluoroscopy times. Furthermore, a study found that fluoroscopy time was reduced 35% (*p* < 0.0001) in the active esophageal cooling group as compared to LET monitoring despite already low levels of fluoroscopy. Joseph et al. also showed that active esophageal cooling maintains a steady 4°C and, compared to only LET, provides a statistically significant improvement from arrhythmia—14% absolute increase in freedom from AF with active esophageal cooling (*p* = 0.03) [6]. Controlling esophageal temperature with cooling devices has shown superiority in reducing esophageal thermal injury [6, 70]. The HEAT‐AF study has shown us that an infrared thermography system allowed the prediction of endoscopically detected thermal esophageal lesions by using peak esophageal temperature. Peak esophageal temperature alone served as an excellent predictor of patients at risk of endoscopically detected esophageal lesions [71]. However, this technique is withdrawn from the market due to several disadvantages such as measuring surface temperatures while inaccurately reflecting intramural tissue temperatures critical for effective lesion formation, high costs, and movement artifacts [72].

#### 6.1.5 Mechanical Esophageal Displacement Increasing the Left Atrium‐Esophagus Distance

The distance between LA and the esophagus is a major determinant of esophageal thermal injury in AF catheter ablation. Maneuvering the esophagus to deflect as far as possible from LA using a deflectable TEE probe or a vacuum suction has been effectively deployed [60, 69]. Aguinaga et al. (2021) showed in a seven‐patient case series that vacuum suction is a safe and promising method for preventing ETI by mechanical deflection of the esophagus ∼30 mm in CB ablation. However, this study is limited to CB ablation [69]. The ESOlution esophageal retractor device is the only FDA‐cleared esophageal manipulation device [73]. EASY AF Trial investigating the use of esophageal deviation in RF ablation showed that there was a significant reduction in ablation‐related esophageal lesions compared to the control group [74] (5.7% vs. 35.4%; *p* < 0.0001). The study was stopped after randomizing 120 patients due to the positive effect of the deviating device. The study also showed that LET monitoring did not predict esophageal ablation injury. On the other hand, improper placement of mechanical esophageal devices may result in increased incidences of mechanical trauma and pharyngeal lesions [75]. Therefore, proper device usage is important to reduce harm to pharyngeal structures [54].

### 6.2 Pulsed Field Ablation: Novel Minimally‐Thermal Tissue‐Selective Ablation Procedure to Reduce Thermal Injuries

#### 6.2.1 Pulsed Field Ablation Technique as a Minimally‐Thermal Novel Technology

Thermal ablation modalities can successfully create scarring in desired ablation zones with high success rates; however, esophageal thermal injuries beginning the cascade leading to esophageal fistula can be a result of conventional thermal ablations. A new minimally thermal ablation technique, ***Pulsed Field Ablation*** (PFA), uses high‐amplitude electric fields to generate irreversible electroporation (IRE) of cardiomyocytes [76]. IRE creates membrane pores and causes cell death [77]. Studies have found comparable efficacy and safe lesions by PFA [78, 79]. In one study, lesions created with pulsed electrical fields raised tissue temperature by a maximum of 2.8°C [80]. The amount of energy to cause cell poration is different for each cell type, and only myocardial tissue‐tailored energy protocols are utilized for PFA, meaning that surrounding structures such as the esophagus, phrenic nerves, coronary arteries, and pulmonary veins are spared [81, 82]. In a recent meta‐analysis by de Campos et al. (2024), including 4998 patients, PFA was associated with lower esophageal injury rates (odds ratio [OR] 0.17; 95% CI 0.06–0.46) and higher tamponade rates (OR 2.98; 95% CI 1.27–7.00) and shorter procedure time (mean difference [MD] –21.68 min; 95% confidence interval [CI] –32.81 to –10.54) but longer fluoroscopy time (MD 4.53 min; 95% CI 2.18–6.88) than thermal ablations [83]. Another recent meta‐analysis by Wamar et al. (2023) included 26 PFA studies with a total of 2561 patients and reported no incidence of AEF nor esophageal injuries [84]. Aldaas et al. (2024) included six comparative studies with 1012 patients who reported no difference in overall periprocedural complication rates when comparing PFA to thermal modalities [85]. No AEF formations were observed, however. Independent studies performing post‐ablation cardiac magnetic resonance imaging also showed no evidence of esophageal injury in the PFA group versus 10% (44) of thermal ablations, even though appropriate esophageal protection (mechanical esophageal deviation, temperature monitoring) was utilized [85, 86]. The meta‐analysis also concluded that PFA was associated with shorter procedural times (MD 5.71, 95% CI 1.13, 10.30, *p* = 0.01) and longer fluoroscopy times (MD –21.95, 95% CI –33.77, –10.14, *p* = 0.0003) with no difference in the recurrence of atrial tachyarrhythmias [85]. Verma et al. (2023) reported no incidence of esophageal injury, pulmonary vein stenosis, or phrenic nerve injury among 300 AF patients undergoing PFA [87]. Thus, PFA is a novel myocardial tissue‐selective minimally thermal ablation modality that may reduce some complication rates and improve the safety and efficiency of AF catheter ablation procedures. Although a standardized anesthesia protocol has not been well‐established, the procedure is preferred to be completed under general anesthesia or deep sedation with anesthesia staff present to lower chest movement caused by chest muscle stimulation, cough, chest pain, and higher operator satisfaction [88, 89]. However, due to the risks of general anesthesia, a deep sedation protocol with a high rate of satisfaction by both the patients and the operators can be implemented [90]. Several other studies practicing similar deep sedation protocols reported similar outcomes compared to general anesthesia [88, 91, 92].

Although PFA offers promising clinical results with an exciting pathophysiological standpoint and freedom from fatal complications, this novel technology is not without downsides. The most common complications with PFA were pericardial effusion, hemolysis, coronary events, and vasovagal responses. When PFA is compared to RF ablation, hemolysis (9% vs. 0%), coronary events (5.8% vs. 0.6%), and vasovagal responses (14.1% vs. 0%) were all reported higher in PFA (*p* < 0.001). Furthermore, catheter malfunction incidences were also reported in a higher proportion after PFA than after RF (87.2% vs. 17.2%; *p* < 0.001) [93]. Future outcome and complication studies with larger and more inclusive population cohorts should demonstrate the actual efficacy and safety of PFA technology. The poor understanding of the cell electroporation mechanism may have multiorgan impairment consequences. The NEMESIS‐PFA study demonstrated that PFA, compared to RFA, was associated with significantly higher troponin levels (13,551.0 vs. 127.5; *p* < 0.001), increased LDH (107.5 IU/L vs. 26.5; *p* < 0.001), greater reductions in haptoglobin (–102.0 mg/dL vs. –33.5; *p* < 0.001), and more frequent renal dysfunction—suggesting that PFA may have more pronounced collateral effects than RFA. The study also concluded that there was a less significant decrease in EF after PFA than after RF ablation (5% vs 20%, respectively).

Moreover, a recent study pointed out that although the technique is minimally thermal, there is a significant temperature rise due to “heat‐stacking” of metal electrodes delivering high‐voltage pulses. The study showed that with irrigation 4 mL/min, baseline temperature increased from an average 22.9°C–61°C, 67.9°C, and 71.4°C on three different successive PFA applications. When irrigated at 30 mL/min, baseline temperature increased from an average 23.2°C–38.5°C, 32°C, and 36.4°C. The study showed that PFA needs external irrigation to prevent excessive temperature rises [94].

#### 6.2.2 Mapping Integration in Pulsed Field Ablation

3D mapping systems have simplified complex catheter ablations, providing feedback tailored to each patient's anatomy and localizing ablation zones. Therefore, mapping integration is important in the treatment of AF. Mapping integration has been recently deployed in PFA, accrediting its wider use [95, 96, 97]. The inspIRE study is the first study integrating 3D‐mapping systems with PFA and successfully reached safety and efficacy endpoints with zero incidence of esophageal lesions as well as nerve injury or pulmonary vein stenosis [98]. All procedures had successful entrance blocks, and PVI without acute reconnection was achieved in 97.1% of the targeted veins. Soon, PFA might replace thermal ablation techniques in atrial tachyarrhythmias [83, 99, 100]. However, larger randomized controlled trials with mapping integration still need to support its safety and efficacy, as well as establish a standardized PFA anesthesia protocol. Finally, PFA has been shown to be well‐suited for ablation and isolation of the left atrial posterior wall, which is in close proximity to the esophagus with favorable safety in comparison to thermal RF and CB ablation modalities for this purpose [101, 102, 103, 104]. Given additional adjunctive posterior wall isolation and lesion sets sometimes require a substantially higher number of PFA applications, future studies are needed to further elucidate how it would be possible to lower adverse events such as coronary vasospasm and hemolysis, as well as tailoring power settings and waveforms [105, 106, 107].

## 7 Conclusion

Atrioesophageal fistula (AEF) is a rare but severe complication of postprocedural AF ablation with documented high mortality rates. Due to their rarity and the lack of related literature, it is often underdiagnosed. Thermal ablation techniques, such as RF and CB ablation, as well as non‐contact force‐sensing catheters, are associated with an increased rate of esophageal injuries and fistula formation. Preventive measures can be preemptively employed, including high‐power short‐duration ablation (50 W for 7–11 s), appropriate sedation based on patient profiles, mechanical esophageal deviation, esophageal temperature monitoring, and cooling devices, and ENPT, although supporting evidence is limited and there is yet no consensus on their true effect on prevention. Water‐soluble contrast chest CT is the modality of choice for fistula diagnosis, and surgery is superior to endoscopic intervention for most patients.

Pulsed‐field ablation is a novel, minimally thermal myocardial tissue‐selective technique that ablates myocardial cells by electroporation. Several meta‐analyses report a zero incidence of esophageal injury using pulsed‐field ablation. However, recent studies demonstrated an increase in temperature around PFA catheters that are well controlled with saline irrigation. Studies also showed increased hemolysis and troponin leak as compared to conventional RF ablation. Thus, larger studies are needed to refine energy settings and prove their safety and efficacy as compared to thermal ablation options. Current literature on high‐risk patient classification remains scarce, highlighting the need for large‐scale prevalence studies to identify risk factors and construct effective prediction tools to minimize AEF occurrence.

## Conflicts of Interest

The authors declare no conflicts of interest.

## Data Availability

The data that support the findings of this study are available from the corresponding author upon reasonable request.
